# Understanding the Unmet Needs among Community-Dwelling Disabled Older People from a Linkage Perspective

**DOI:** 10.3390/ijerph18020389

**Published:** 2021-01-06

**Authors:** Danxian Wu, Xiaolu Gao, Zhifei Xie, Zening Xu

**Affiliations:** 1 Key Laboratory of Regional Sustainable Development Modeling, Institute of Geographic Sciences and Natural Resources Research, Chinese Academy of Sciences, Beijing 100101, China; 2 University of Chinese Academy of Sciences, Beijing 100190, China; xiezhifei15@mails.ucas.ac.cn; 3 Institute for the History of Nature Science, Chinese Academy of Sciences, Beijing 100101, China; 4 China Institute for Urban Governance, Shanghai Jiao Tong University, Shanghai 200030, China; xuzn.13b@igsnrr.ac.cn

**Keywords:** unmet needs, linkage, disabled older people, elderly care, enabling environment, community-based care, inequality

## Abstract

One of the challenges in response to population aging is to meet needs for elderly care among older people especially for those who want to age in their homes or communities. However, disabled older people have more challenges due to their restricted mobility to access care resources than non-disabled ones. We propose a new framework based on the changing relationship between older people and their environment, in which resource linkage in elderly care utilization is emphasized. We conducted a survey with 139 participants (i.e., older people age 60 years or over with different level of disabilities) in three types of neighborhoods in Beijing, China. By conducting a decision tree analysis under the Person-Environment Link (P-E Link) model, we (1) characterized unmet needs for elderly care (activities of daily living (ADL) and instrumental activities of daily living (IADL) assistance) among community-dwelling disabled older people; (2) found disabled older people had more unmet needs for both ADL and IADL assistance because of a lack in linkages to care resources than non-disabled ones; and (3) characterized the linkages to care resources for better supporting disabled older people to age in place, including family support, social connection, and spatial environment. Our findings help improve the Anderson behavioral model by characterizing enabling environments, which highlights that not only the availability of enabling resources but also linkages to these enabling resources play an important role in meeting needs for care among disabled older people. Our findings can also inform improvements in policy design that are targeted to reduce elderly care inequalities.

## 1. Introduction

As population ages, the contradiction between the surging care needs of disabled older people and the shortage of care resources has become a global problem. Failure to obtain sufficient assistance creates unmet needs among disabled older people. In developed countries, having unmet needs has become a common problem with deinstitutionalization across a range of care services [1,2,3,4]. Notwithstanding the differences in welfare systems, there is congruence across the fact that unmet needs continue to escalate among disabled older people in developing countries where traditional informal care dominates without a non-institution reform of care services [5,6]. Policy reform initiatives that aim to keep older people out of institutions and in their own homes or communities for longer have been at the forefront since the 1980s [7,8,9]. The change in the living arrangements of older people has led to worse inequity and inequality in elderly care utilization, and thus the growing prevalence of unmet needs [10,11,12,13,14]. In many instances, community-dwelling disabled older people who have difficulty performing basic activities of daily living (ADL) or instrumental activities of daily living (IADL) suffer more due to their vulnerability and restricted mobility [15,16]. 

Evidence from recent research shows that as needs for elderly care increase and public spending tightens, the unmet needs among disabled older people grows [1,4]. However, it is apparent that no unanimous conclusion can be drawn in the causes or correlating research, despite the consensus on the prevalence of unmet needs among disabled older people. A group of studies have emerged as the common theme of modelling the key feature of socio-demographic characteristics, service characteristic, and other factors in an attempt to explain and predict unmet needs [17,18]. Much of the research in this area has been guided by the Andersen behavioral model emphasizing predisposing factors, illness level, and enabling factors [19,20].

However, most of the research represents more of an attempt to specify the nature of variables influencing utilization rather than a clarification of key factors targeted toward disabled older people. They take for granted that disabled people share the same pattern with others when it comes to elderly care services utilization. They see older people as a homogenous group, and thus the understanding of unmet needs among different subgroups is ambiguous [21]. Perhaps the greatest stumbling block to effective application of this type of model is that, as in the majority of models, the normative assumptions upon which they are based limit their explanatory power in the dynamic process of unmet needs among disabled older people. For the policy makers and health service planners, it is a problem that has for so long hampered the improvements of elderly care delivery system. With gradual spatial constriction that occurs as people age, older people change the way they interact with the environment. Behavior is restricted or enhanced by environmental characteristics and individual functional capacity [22]. Physical limitations shape utilization behavior, health outcomes, and consequently change the quality of life [23,24]. There seems to be a growing need for a new conceptual framework to understand the complex phenomenon of unmet needs of disabled older people and to guide empirical research. It is, therefore, necessary to concentrate on disabled older people as a group with particular environmental vulnerabilities and a strong need for resource linkage, particularly in the elderly care context.

With a view to filling this identified gap, this paper aims to explore the factors influencing unmet needs for elderly care based on a new framework named Person–Environment Link (P-E Link) model. In particular, we are interested in knowing whether the inadequate linkage to care resources may affect the unmet needs for elderly care among disabled older people. This study would contribute to what can be regarded as an extension of research on aging by better understanding how the unmet need can be identified in diverse aging community-dwelling people. It would also provide implications for policymakers to reduce health inequalities and narrow the gap between the vulnerable older population and others.

The remainder of the paper is organized as follows. Beginning with a discussion of theory development and the conceptual gap identified, the next section proposes a new framework, in which resource linkage in elderly care service utilization is emphasized. Subsequently, a case study is carried out to explore the unmet needs of disabled older people through empirical studies of three types of neighborhoods in Beijing, China. The study utilizes decision tree analysis to identify the key factors affecting satisfaction in elderly care service utilization. Finally, the implications and limitations of this study are discussed.

## 2. Conceptual Framework 

### 2.1. Limitations in the Literature

A large body of research on health care utilization exists but elderly care utilization among older people with varying degrees of disability has seldom been explored. Andersen behavioral model of health services utilization is usually applied to study elderly care services utilization and explore the dynamics of unmet needs among older people [19,25]. This popular model originated from health care behavior research and is frequently used to describe how a need for health care “can” or “can’t” be translated into access [26,27]. However, the model has lost some of its impact with researchers who focus on disabled older people as a group with particular environmental vulnerabilities and a strong need for optimized environments.

Firstly, the research based on Andersen’s Behavioral Model has focused more on medical care than elderly care which disabled older people exactly need. Medical care, a service people of all ages can use, is a professional service with the goal of recovery, and where it happens tend to be health care facilities like community health centers or hospitals. By contrast, elderly care including ADL and IADL assistance to maintain an autonomous life, is usually a series of services. It can occur in homes, communities, assisted living institutions, nursing homes, and other places.

Secondly, the research adopts a physician- or facility-oriented approach instead of client-oriented one. They focus on one kind of facilities like hospitals, nursing homes, or pharmacies, taking accessibility as an important geographic factor. There are, however, some problems in these models such as simplification, for the reason that it is impossible to study the diverse and multiple needs of disabled older people based on one particular kind of facility. Elderly care, as we know, is a series of services, so the need for it among disabled older people is achieved through the use of different and diverse facilities rather than a single one.

Thirdly, the factors explored in these studies are numerous non-geographical determinants, so that it is insufficient to explain why some needs among community-dwelling disabled older people are not met. Although some studies have taken geographic factors into account, there are still gaps. For the most important geographical perspectives of potential access, researchers have used two basic options to measure access: a "regional availability" approach and a "regional accessibility" approach [28] in the past. For disabled older people with limited mobility, there is often a problem of geographical scale failure. Some studies measure accessibility among older people at a community scale [29,30]. However, declining physical capabilities and increasing effort to maintain a geographically extensive activity space, as well as the loss of significant social roles (e.g., retirement and empty nest) and the subsequent loss of economic income, means that disabled older people tend to spend more time at home and their ability to overcome space is limited by their health status and material conditions [31,32]. Good physical accessibility does not necessarily mean efficient utilization among disabled older people.

### 2.2. Key Concepts in the Elderly Care Utilization Context

The decline of physiological functions is the most significant and essential feature that distinguishes disabled older people from other groups. It is well-established that declines in physiological function lead to declines in individual mobility, which corresponds to the declining physical accessibility to service facilities. In this way, the solution to reduce unmet needs is to improve accessibility. To achieve this goal, the construction of community facilities and service-enriched housing is widely advocated [33]. However, there is a lot of evidence that the effectiveness of these measures is far from satisfactory [34,35,36]. Accessibility is only a necessary but not sufficient condition for service utilization. From this perspective, accessibility is very limited in understanding unmet needs of older people with disabilities.

No one can deny the effects of spatial constraints on disabled older people, which is a significant characteristic of the relationship between older people and their care environment. This is not only manifested in the decline of mobility of older people, but also in a series of changes such as shrinkage, fragmentation, and transfer of the environment in which they live. Staying at home because of disabilities necessitates abandonment of a familiar mode of being in place. In other words, it requires abandoning familiar patterns in the interaction between older people and their surroundings.

The change in the relationship between a person and their environment means a change in the pattern of utilization of care services among disabled older people. Specifically, their pattern of taken-for-granted use of space changes as they grow older and become frail, which is different from non-disabled groups. The needs of disabled older people cannot be met only by money or material construction, but they need external help and the delivery of care services. Therefore, we assume that for disabled older people, independence is more important than illness level; for the environment, the care environment based on the daily life experience is more important than the facilities; and for the person-to-environment relationship, the link to a broader set of resources is more important than the direct utilization of facilities or services. In sum, we argue that linkage to resources is more important than accessibility to facilities for disabled older people [37], especially for those who want to age in their homes and communities.

To support our assumptions and arguments, a new conceptual framework is proposed to provide deeper insight into the unmet needs of disabled older people based on these key concepts.

### 2.3. A New Framework

**Figure 1:**
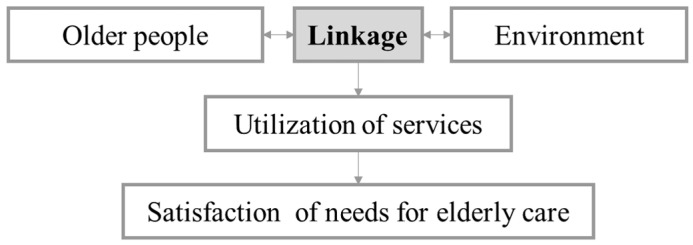
Conceptual framework on satisfaction of needs for elderly care from a linkage perspective.

The conceptual basis for this research draws on the P-E Fit model developed by Lawton and Nahemow [38]. The new framework in this study also relies on the transformation of person-environment interaction pattern when older people become disabled, in which resource linkage is emphasized. Under this model, the goal is to find the “best fit (linkage)” between the component “older people” and the component “care environment”. Optimal performance (utilization of services) and outcome (satisfaction of care needs) occur when an optimal balance is achieved between the person’s needs and the environment (Figure 1). These five components of the model are dynamic and constantly changing. The balance between individual competence (need) and environmental resource (supply) is constantly evolving as either the individual’s competence or the supply of the environmental resource changes. The models push one to think about how older people really live but not focus only on the remediation of their health problems.

The above basic model can be used to explain how unmet needs among older people come into being. The dynamics of unmet needs in the utilization of elderly care is the maladjustment between older people and their care environment, and thus the failure of effective linkage to care resources (namely inadequate linkage). That might be due to a chronic health condition or financial, educational, and social disadvantages, but more research is needed to uncover the mechanisms by how those factors translate into barriers to utilization.

**Figure 2:**
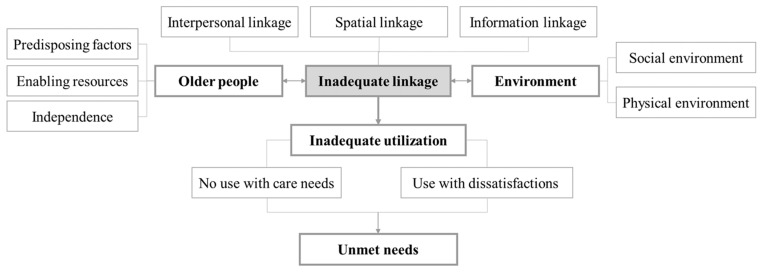
Person-Environment Link (P-E Link) model for unmet needs for elderly care.

More specifically (Figure 2), individual factors (the component “older people”) are divided into predisposing factors, enabling resources and independence. Environmental factors are external influences on service utilization that include features of the social and physical environment. Linkage between older people and their care environment, includes interpersonal linkage, spatial linkage and information linkage. “Unmet needs” occur when disabled older people fail to get help or do not get enough help to perform their daily tasks. Inadequate linkage can lead to inadequate utilization of care services. In this case, there will be two situations in which older people who have needs do not use services (“no use with care needs”) and older people who use a service but are not satisfied (“use with dissatisfactions").

## 3. Materials and Methods 

### 3.1. Data

Beijing is a megacity leading the high-speed ageing process with the largest number of older people in China. By the end of 2018, the number of older people was about 3.44 million and the total number of disabled older people exceeded 150,000 in Beijing [39]. Financial barriers to care are more likely to be addressed by financial support for elderly care and social security coverage for older people from the government in Beijing than from other private or public sources. Selecting Beijing as the study area not only reduces the masking effect of economic constraints over other factors, but also has a practical significance for exploring the future direction of elderly care delivery systems in China.

Taking into account that disabled older people are sparsely distributed throughout the Beijing region and study resource limitations, we conducted a survey in three neighborhoods in Beijing. Taking into consideration the spatial distribution, environment, and care services, we selected the three neighborhoods as representative of different “types” (traditional courtyard housing block, work unit compound, and mixed community) of typical neighborhoods. Twelve graduate students underwent training and a 3-day pre-survey practice session prior to commencement of the study to ensure that every investigator was qualified in his or her work. Face to face interviews were conducted at participants’ homes or community centers from November 2018 to December 2019.

A cohort of 150 older people were recruited for the study designed to identify the risk factors for unmet needs in older community-dwellers. Eligibility criteria for the study were: age 60 years old and above; living at home; at various disability levels; having the ability to answer questions or have caregivers who are familiar with the situation to answer questions. Older people with intellectual disabilities were excluded from this study. The surveys resulted in a valid sample of 139 people.

Rows of “Personal attributes of older people” Part in Table 1 summarizes the characteristics of the participants. Participants were mostly aged 80 years and above, female, widowed, graduated from elementary or middle school. Nearly 70 percent of the Participants had more than one children and had family monthly incomes under 10,000 Yuan. According to ADL and IADL scales they varied in the level of disabilities. They preferred to stay at home or the community due to restricted mobility.

### 3.2. Ethics

This study was approved by the Ethics Committee of the Institute of Geographic Sciences and Natural Resources Research, CAS (Project identification code: 20180918001) and was carried out following the principles of the Declaration of Helsinki. The study participants were informed of the purpose of the study and assured of keeping their identity and responses confidential. Refusal to participate or to discontinue participation at any time was allowed.

### 3.3. Dependent Variables

**Figure 3:**
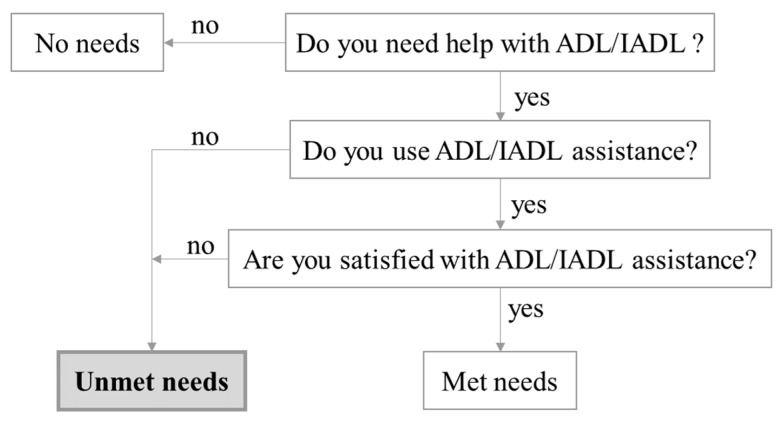
Determination of unmet needs for activities of daily living (ADL)/instrumental activities of daily living (IADL) assistance.

In this study, whether there were unmet needs for elderly care (ADL and IADL assistance) was defined as the dependent variable. Unmet Needs Assessment was based on a tool that was adapted from an algorithm created by Allen [40] and modified by Chen [41]. Using this tool, unmet needs were identified according to three questions (Figure 3): (1) Do you need help with an ADL or IADL assistance; (2) Do you use ADL or IADL assistance; (3) Are you satisfied with ADL or IADL assistance. Investigators conducted interviews with disabled older people following the guidelines of this assessment. If a respondent who has needs for elderly care did not use ADL or IADL assistance or used the assistance but was not satisfied with it, the respondent was considered a valid case with unmet needs. Katz Index of Independence in Activities of Daily Living is an appropriate instrument to assess functional status as a measurement of the client’s need for ADL assistance. Clients are scored yes/no for independence in each of the six functions, including bathing, dressing, toileting, transferring, continence, and feeding [42]. The Lawton Instrumental Activities of Daily Living Scale is an appropriate instrument to assess independent living skills as a measurement of the client’s need for IADL assistance [43]. Clients are scored according to their highest level of functioning in a category with 8 domains of function.

### 3.4. Independent Variables

Following the new framework P-E Link Model, the decision tree analysis for unmet needs in this study take into account the following factors: personal attributes of older people, environmental characteristics and their interrelationships, namely, linkage.

The personal attributes of older people included age, gender, marriage, educational background; monthly income, the number of children, internet utilization; ADLs, IADLs, and mobility. These variables can be respectively grouped into three broad categories: predisposing characteristics, enabling resources, and independence.

Environmental characteristics included neighborhood type, seen as the variable that embodied the social environment; density of community facilities; and residential housing area seen as the variables that embodied physical environment.

Linkage included living arrangement, the number of close friends, the number of close children; distance of the nearest facility, distance of the nearest children; and service hotline utilization. These variables can be grouped into three broad categories: interpersonal linkage, spatial linkage and information linkage.

Table 1 provides an overview of the data used, the variables analyzed, and the distribution of the studied population over these variables.

### 3.5. Statistical Analysis

We used JMP13.3 software to perform decision tree analysis to validate our theoretical framework. Decision tree analysis is an effective method for data mining and classification. It can not only classify the target variable into several subgroups of a tree structure, but also effectively analyze interactions between continuous variables and discrete variables [44,45]. We established two models respectively for ADL and IADL assistance

Decision tree analysis was conducted for element identification rather than prediction in this study. The depth of the tree was set at two levels, and the minimum number of cases of the node was set to five. The model stopped when two conditions were met: (1) column contribution value of the new variable was below 0.05; (2) R^2^ growth slowed down significantly. In this way we identified the variables that played a major role in the dynamics of unmet needs among disabled older people.

## 4. Results

**Figure 4:**
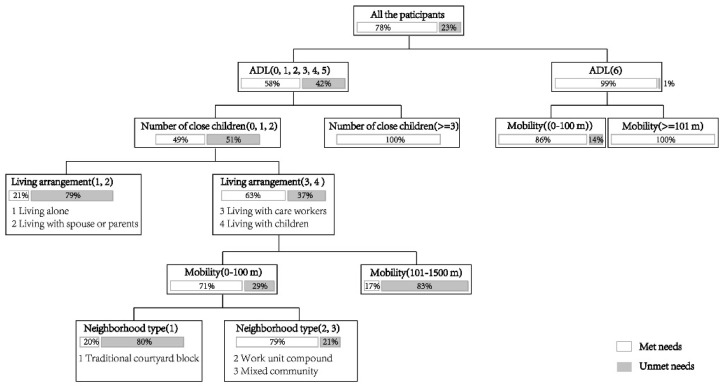
Decision tree for unmet needs for ADL assistance. (Note: The gray bars represent unmet needs, and the numbers represent the percentage).

**Figure 5:**
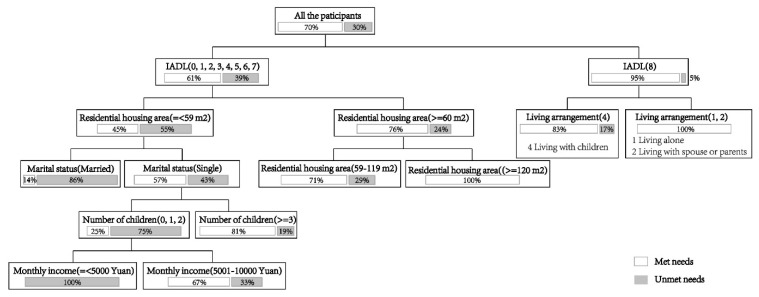
Decision tree for unmet needs for IADL assistance. (Note: The gray bars represent unmet needs, and the numbers represent the percentage).

Results from the statistical analysis are shown in the following tables and figures. Table 2 and Table 3 shows the results from descriptive statistical analysis of unmet needs, Figure 4 from the decision tree analysis for the ADL model and Figure 5 for the IADL model.

### 4.1. Prevalence of Unmet Needs

The results showed that 63.31 percent of the older people had their needs for ADL and IADL assistance met at the same time, but still more than one-third of the participants had at least one unmet need. Among the participants with unmet needs, there were 15.8 percent who had needs for both ADL and IADL assistance, 6.5 percent only for ADL assistance and 14.4 percent only for IADL assistance (Table 2).

Both disabled and non-disabled older people who needed a service experienced access-to-care barriers and thus had unmet needs. Nearly 40 percent of non-disabled older people had unmet needs only for IADL assistance, while more than 40 percent of disabled ones had unmet needs for both ADL and IADL assistance (Table 3). In other words, disabled older people were more likely to have unmet needs for elderly care, which resulted in a greater risk of quality of life decline.

### 4.2. Factors Influencing Unmet Needs for ADL Assistance

According to R^2^ changes and column contribution values, the ADL model stopped when there were six branches with 0.548 R^2^ value. Among the variables, “independence” (ADL and mobility) had the largest direct effect on unmet needs, followed by “interpersonal linkage” (the number of close children and living arrangement) and “social environment” (neighborhood type). The top five variables also explained most of the model variance. The results imply that independence, interpersonal linkage and social environment were crucial to ADL assistance utilization among older people, especially those in a disability status.

Independence: As expected, ADL was the most significant predictor in the ADL model. Disabled older people whose ADL score was below six had a much higher probability of unmet needs for ADL assistance than non-disabled older people. For non-disabled older people, those who just stayed at home had more unmet needs than those who had better mobility. However, for disabled older people, those who just stayed at home had less unmet need than those who had better mobility. It is clear that mobility has different effects on older people with different degrees of disability.

Interpersonal linkage: The factor, less than three close children (who were in frequent contact), greatly increased the probability of disabled older people with unmet needs for ADL assistance. The number of close children, instead of the number of children, played an important role in the use of any ADL assistance. Among the disabled older people, living alone or with his or her spouse was associated with an increased likelihood of unmet needs for ADL assistance.

Social environment: The disabled older people living in the traditional courtyard block had a higher prevalence of unmet needs for ADL assistance.

### 4.3. Factors Influencing Unmet Needs for IADL Assistance

According to the R^2^ changes and column contribution values, the IADL model stopped when there were seven branches and the R^2^ value was 0.395. In general, the IADL model was less powerful than ADL model and the influence of factors was more dispersed. Among the variables, “enabling resources” (the number of children and income) had the largest direct effect on unmet need, followed by “independence” (IADL), “physical environment” (residential housing area), “predisposing factors” (marriage) and “interpersonal linkage” (living arrangement). The results imply that enabling resources, independence, physical environment, predisposing factors and interpersonal linkage were important to IADL assistance utilization among older people, especially those with a disability.

Enabling resources: Having less than three children greatly increased the probability of disabled older people with unmet needs for IADL assistance. A higher monthly income was associated with a decrease in the prevalence of unmet needs for IADL assistance.

Independence: IADL score was a significant predictor for the prevalence of unmet needs for IADL assistance. Disabled older people had a much higher probability of unmet needs than non-disabled ones.

Physical environment: For disabled older people, those who lived in a smaller apartment had an increased likelihood of unmet needs for IADL assistance.

Predisposing factors: Disabled older people who were married suffered more from unmet needs for IADL assistance than those who were unmarried, widowed or divorced. This finding is different from many previous studies. This is because married older people tended to live only with spouses who are also in their old age, while those in other marital states were more likely to live with younger caregivers and experience fewer barriers to care.

Interpersonal linkage: Among non-disabled older people, living alone or with his or her spouse (also in old age) was associated with a decreased likelihood of unmet needs for IADL assistance.

## 5. Discussion

### 5.1. Individual Ability and the Environment 

In line with other studies [46,47,48], our study finds that disabled older people are the main group suffer from the unmet needs for elderly care. Further to this, the division of different subgroups of older people based on individual ability are needed to be discussed. It was suggested that older people could be categorized into more than two groups allowing caregivers to plan and intervene appropriately [49]. For example, the original grading is adapted to a trichotomy of full function, moderate impairment and severe impairment according to ADL scores in practice [50]. However, the breaking point of the branch lines is in the 5–6 score range for the ADL model and in the 7–8 score range for the IADL model in our study. The results of dichotomy among older people questions the guidelines for using the assessment tool to differentiate older people. 

Individual ability (measured by ADL or IADL scale) is only one of the factors that will determine what an older person can do, though it distinguishes the older people with full function from others when it comes to the fulfillment of needs for elderly care. For those who has problems in at least one function (collectively referred to as disabled older people in our study), the other is the environments they inhabit and their interactions with them. For example, a disabled older person having a less score of ADL score may maintain higher levels of functioning and greater satisfaction with accessing care services when living in a more age-friendly community [31]. It means that unmet needs for care among those older people having mild impairment partly result from a poor environment.

The association between individual ability and unmet needs is neither linear nor absolutely consistent. Therefore, it is unreasonable to organize care resources only taking one’s individual ability into account. In terms of reducing health inequalities, one action advocated by WHO is to enable greater functional ability in someone with a given level of intrinsic capacity [31]. In other words, filling the gap between what people can do given their level of capacity and what they could do if they lived in an enabling environment (for example, by providing appropriate assistive technologies, providing accessible public transport or developing safer neighborhoods) would reduce the level of unmet needs. Therefore, barriers to utilization of care services should be removed and loss of ability of disabled older people should be taken into account in order to reduce unmet needs.

### 5.2. Mobility and Access to Elderly Care 

Consistent with findings from the previous studies [41,51,52], we confirm the importance of predictors, such as old age and health status, living alone, and living in a rented house, as contributors to elderly care service utilization. Previous literature has shown that older people with worse mobility has less access to health care, suffering more from the unmet needs [53].Therefore, our finding of a positive association between mobility and unmet needs among disabled older people but a negative association among the non-disabled group was unexpected. One possible explanation is that the two groups have different ways of accessing elderly care services. Disabled older people access services relying on their caregivers instead of themselves. In contrast, the non-disabled older people obtain the services they need independently, in which the maintenance of mobility is thought to be fundamental. 

For non-disabled older people, mobility plays the most important role in receiving ADL assistance. In this sense, accessibility as a geographic factor should be a priority issue in care resources organization. However, the dynamics of unmet needs among disabled older people is more complicated. For example, accessibility factors such as spatial distance and density of facilities are not identified as key factors in our study compared to some literature [29,30]. A comprehensive policy response should be able to reconcile these different emphases into a coherent narrative of ageing [31].

### 5.3. Linkage in the Care Delivery for Disabled Older People 

Our study highlights that older populations are characterized by great diversity. Indiscriminately meeting the care needs of such diverse populations can result in policies that appear disjointed and inefficient. In this respect, the huge difference between the disabled and the non-disabled needs to be addressed. However, Anderson behavioral model failed to give a deeper insight into neither the disabled older people nor their interactions with the environment [27,54]. Therefore, we argue that the dynamic process of unmet needs among disabled older people with particular environmental vulnerabilities and a strong need for resource linkage are best explored under a new framework. P-E Link model we propose emphasize adequate linkage to care resources in the process of changing the relationship between disabled older people and their environment. Our study is a better illustration of the differences between the disabled and non-disabled older people.

On the other hand, our findings help improve the Anderson behavioral model by characterizing enabling environments, which highlights not only the availability of care services but also the age-friendliness of communities. The construction of enabling environments at the community level requires not only the construction of health care facilities, but also the effective linkage to care resources in order to optimize the care delivery for disabled older people. In this regard, policy makers should support disabled older people and their caregivers in becoming aware of the necessity of a stable linkage to resources in their surrounding environment. 

## 6. Conclusions

In this study, we propose a new framework for understanding and analyzing the unmet needs of community-dwelling older people, especially for those having functional disabilities. Basically, people of all ages experience an environment through its use. In the P-E Link model, the fulfillment of a person’s need is viewed as a dynamic interaction, namely linkage, between the individual and the environment. We also provide empirics that older people do not receive help to access elderly care and further substantiates factors associated with the unmet needs for elderly care. The results of the analysis reveal the important role of the person–environmental linkage in reducing the unmet needs for ADL assistance and the important role of enabling resources in reducing the unmet needs for IADL assistance among disabled older people.

The study makes a contribution to understanding how unmet needs can be identified in diverse aging people differentiating the dynamics of unmet needs between disabled older people and non-disabled ones. We hope that this study may enrich the existing knowledge on the interaction between older people and their environment and provide a reference for policy makers.

There were some limitations in the study which may provide avenues for future research. The primary limitation concerns cross-sectional nature of the study with a small-size sample, which makes the establishment of causal links methodologically difficult. Another limitation arises from the fact that all data were collected using the self-reporting or surrogate report method. This may lead either to over-estimation or under-estimation of the variables. Still, given that this study was intended to be exploratory, and that appropriate frameworks for older population characterized diversity are still scarce, our analyses nevertheless provide preliminary information on unmet needs for elderly care among disabled older people.

## Figures and Tables

**Table 1:** Variables in P-E Link model for decision tree analysis.

Factors		Variables	Level	Proportion
**Personal attributes of older people**	**Predisposing factors**	Age	60–69	9%
			70–79	25%
			80–89	47%
			>=90	19%
		Gender	Male	35%
			Female	65%
		Marital status	Married	39%
			Single (unmarried/divorced/widowed)	61%
		Educational background	Elementary school and below	41%
			Middle school	27%
			High school	14%
			Junior College and above	18%
	**Enabling resources**	Monthly income (Yuan)	<2000	7%
			2001–5000	29%
			5001–10,000	34%
			>10,000	30%
		The number of children	0	9%
			1	19%
			2	33%
			>=3	39%
		Internet utilization	Good	9%
			Fair	14%
			Poor	77%
	**Independence**	ADL	0–1	18%
			2–3	10%
			4–5	24%
			6	48%
		IADL	0–1	29%
			2–4	25%
			5–7	19%
			8	27%
		Mobility (meters)	0–100	47%
			101–500	26%
			501–1500	12%
			>1500	14%
**Environments characteristics**	**Social environment**	Neighborhood type	Traditional courtyard block	32%
			Work unit compound	29%
			Mixed community	39%
	**Physical environment**	Density of community facilities (per square kilometer)	Elderly care facilities for ADL model	
			Retail outlets for IADL model	
		Residential housing area (square meters)	=<59	47%
			60–99	27%
			100–119	14%
			>120	12%
**Linkage**	**Interpersonal linkage**	Living arrangement	Living alone	22%
			Living with spouse or parents	24%
			Living with care workers	14%
			Living with children	40%
		The number of close children	0	25%
			1	38%
			2	22%
			>=3	15%
		The number of close friend	0	38%
			1	13%
			2	17%
			>=3	32%
	**Spatial linkage**	Distance of the nearest facilities (meters)	Elderly care facilities for ADL model	
			Retail outlets for IADL model	
		Distance of the nearest children (meters)	>=0	63%
			No children	7%
	**Information linkage**	Service hotline utilization	Good	7%
			Fair	31%
			Poor	62%

ADL: activities of daily living. IADL: instrumental activities of daily living.

**Table 2:** The satisfaction of needs for ADL and IADL assistance among all the participants.

		IADL	
		Met Needs	Unmet Needs
**ADL**	Met needs	63.31%	14.38%
	Unmet needs	6.47%	15.83%

**Table 3:** The proportion of disabled and non-disabled older people with unmet needs.

	Unmet Needs for ADL	Unmet Needs for IADL
disabled older people	41.67%	40.28%
non-disabled older people	1.49%	19.40%
